# A Multipronged, Community-Partnered Intervention (The TALK) to Improve Parent-Adolescent Communication About Sexual Health and Racial Discrimination Among Black Male Adolescents and Young Adults and Their Caregivers: Protocol for a Feasibility and Acceptability Study

**DOI:** 10.2196/67403

**Published:** 2025-07-08

**Authors:** Schenita D Randolph, Ragan Johnson, Maralis Emerson, Jolie S Jemmott, Elizabeth Jeter, Allison Johnson

**Affiliations:** 1 Duke University School of Nursing Durham, NC United States

**Keywords:** parent-adolescent communication, sexual health, STI and HIV prevention, eHealth, mixed methods, community engagement

## Abstract

**Background:**

Unsafe sexual behaviors among Black male adolescents and young adults increase their susceptibility to negative health outcomes that widen persistent health disparities. Parent-adolescent relationships and communication can impact Black male adolescents and young adults’ sexual health behaviors, but parents and adolescents often lack knowledge and effective tools to improve health outcomes. Culturally tailored sexual health interventions that integrate the intersectionality of race, gender, family, and social influences on sexual health are limited yet needed to reverse these trends.

**Objective:**

This project aims to develop a nurse-led multipronged intervention, The TALK, which is a parent-centered, adolescent-involved health promotion intervention for Black male adolescents and young adults.

**Methods:**

This mixed methods study uses a community-engagement approach to develop and pilot a parent-centered eHealth intervention. There are 3 research phases: development, usability for community of interest, and testing for real world usability. First, The TALK development is tested with parents and caregivers by using the dscout platform, a digital platform for virtual ethnographic research used to explore early-stage user experience. Second, we will recruit parent-adolescent dyads for a pretest-posttest survey data collection to examine the usability, acceptability, and preliminary intervention outcomes. This phase focuses on the frequency and quality of parent-adolescent sexual health and racial discrimination communication, improvements in knowledge of HIV testing, improvements in parent-adolescent conversations around racial discrimination, and its impacts on sexual health and improved perceived racial identity among Black adolescents. Third, we will examine the usability of the intervention’s web-based modules through promotion in real-world settings of barbershops and beauty salons across North Carolina through signage (with a QR code to scan and access the website). We will measure the usability through website metrics, including page views, average time on page, average session duration, pages per session, bounce rate, and traffic sources.

**Results:**

This project was funded by the Gordon and Betty Moore Foundation and approved by the Duke University School of Nursing institutional review board in September 2022 (Pro00105116) for development with the community. Intervention components were developed in partnership with community partners in the first year. Data collection for phase 1 began in October 2022. Data collection for phase 2 began in July 2023 and ended in January 2024. Data analysis is scheduled for completion by July 2025. The primary and secondary results are expected to be published by January 2026.

**Conclusions:**

Culturally tailored interventions that include content on the intersectionality of race, gender, and family and social relationships combined with strategies to improve parent-adolescent communication have promise for promoting sexual health and racial identity among Black male adolescents and young adults. Our findings have the potential to influence intervention design and research for other populations and contribute to broader efforts to reduce health disparities.

**International Registered Report Identifier (IRRID):**

DERR1-10.2196/67403

## Introduction

### Background

Black male adolescents and young adults aged 13-24 years are 13 times more likely to acquire HIV infections compared to White male adolescents and young adults, accounting for over half of all the HIV infections in this age group in the United States, with additional disparities in the Southern United States [1-3]. Racism, sociopolitical drivers of health, homophobia, and other longstanding barriers are the key drivers to disparities in HIV prevention, care access, and treatment among Black male adolescents and young adults. Without culturally relevant, effective prevention and care interventions, new adolescent infections among Black males are projected to rise by 13% annually, leading to 3.5 million new infections by 2030 [4]. Combating the HIV epidemic among Black male adolescents and young adults requires strategies that emphasize culturally tailored messaging that prioritize their needs and concerns and integrate important adult social influences such as parents and trusted community members (ie, barbers and stylists) [2].

Among the many contributors to HIV disparities, racialized discrimination affects Black male adolescents, leaving them increasingly vulnerable to HIV [5]. Studies have shown that racial discrimination and perceived racial identity are linked to sexual behaviors that increase HIV vulnerability, including inconsistent and/or condomless sex, multiple sexual partners, inconsistent condom use, and substance abuse before sex [6-9]. Black families play a crucial role in influencing how adolescents understand the meaning of race. Self-affirmation activities, parental messaging, and parent-adolescent conversations about racism and perceived racial identity early in adolescence have the potential to have a current and long-lasting impact on sexual health decision-making [10-15].

Parental engagement of Black parents in behavioral HIV prevention interventions has been found to be effective in delaying sexual debut and promoting condom use among male adolescents [16,17]. However, only a few interventions have kept up with the ever-evolving structure of the family; these interventions have mainly engaged fathers and sons or mothers and sons, and limited interventions have engaged Black fathers [16,17]. What is missing from adolescent sexual health interventions for Black male adolescents and young adults is an inclusive approach that engages both the mother and father, regardless of their marital or residential status (ie, living in or outside of the home) and interventions that are culturally and socially aligned with the lived experiences of community members. Furthermore, current interventions for adolescents are delivered primarily through face-to-face delivery or provider and facilitator-led programs, and although these interventions have shown some level of effectiveness, the need for multipronged, community-partnered interventions are warranted to improve sustainability over time.

Given the multiple social, structural, and political determinants of health affecting Black youth, it is imperative that interventions use a multipronged approach to address interconnected issues and maximize impact [18]. Multipronged strategies can provide a more comprehensive solution through interventions that are responsive to the needs and experiences of Black youth and engage community partners [19]. Barbershops and salons have a long history of being a unique, trusted, and effective space for health promotion, specifically for Black communities [20,21]. By regularly engaging with fathers, mothers, and their sons, barbers can facilitate and normalize conversations around sexual health norms and serve as a distribution point for sexual health materials such as digital and print educational media.

Overall, the availability of effective behavioral interventions for adolescents that address the unique circumstances and relationships they face as well as those utilizing technology are limited. Technology-integrated interventions are needed to keep up with the social changes of engagement [16]. Technology-delivered family interventions that include father and/or mother have the potential for widespread community reach and are urgently needed [22]. To our knowledge, there are no technology-delivered adolescent sexual health intervention strategies that address racial discrimination and perceived racial identity. In response, our intervention, The TALK, was co-developed with researchers, clinicians, barbers, and community members to improve parent-adolescent communication of sexual health and experiences of discrimination and racism.

### Theoretical Framework

The TALK is grounded in the syndemic theory [23,24] and uses a social ecological framework to address the health needs and inequities of Black communities through a holistic approach that accounts for multiple nested levels of factors that contextualize health outcomes—from the individual to the structural [25,26]. In other words, the framework attempts to capture the complex interconnection and influence of multilevel determinants that help health researchers understand and explain Black health inequities, including the ways in which challenges such as stress function simultaneously with positive resistance, like resilience and other assets, improve the health of Black communities, especially around chronic illnesses such as HIV infections [27]. By combining the syndemic theory with a social ecological model, this study not only identifies the co-occurring and interacting conditions (eg, HIV risk, racial discrimination) but also situates them within a multilevel context that includes individual behaviors, family dynamics, community norms, and structural inequities. This integration allows us to design an intervention that is both theoretically grounded and practically responsive to the lived realities of Black male adolescents and young adults. The social ecological framework broadens the research lens with an intersectional and intersectoral approach for studying the experience of sexual health and HIV in Black communities [24,27,28]. To understand HIV infections among Black male adolescents and young adults, we must account for how structural, social, behavioral, and biological factors operate to create a context in which Black male adolescents are particularly vulnerable to acquire HIV. A syndemic approach describes how the co-occurrence and interactions between 2 or more conditions can disproportionately affect the health and well-being of marginalized communities [23,24]. The syndemic model [27] conceptualizes HIV transmission risk and racial discrimination as tightly interwoven and interacting public health issues contributing to HIV disparities among Black male adolescents and young adults. At each corner of the model are risk or protective factors associated with syndemic conditions. Adapted from ecological models [25,26], these factors are categorized into individual, interpersonal, cultural, and socioenvironmental levels of influence. Unlike traditional ecological models, culture is identified as a separate level of influence to underscore its importance in shaping health behaviors and outcomes.

The Talk intervention operationalizes the social ecological model by embedding targeted strategies at each level of influence. At the individual level, it enhances adolescents’ knowledge of HIV prevention and fosters a stronger sense of racial identity. At the interpersonal level, the intervention strengthens parent-adolescent communication about sexual health and racial discrimination, aiming to improve both frequency and quality of these critical conversations. The cultural level is addressed by promoting positive racial identity and incorporating culturally affirming messages that resonate with the lived experiences of Black families. At the community level, the intervention engages trusted spaces of barbershops and beauty salons to disseminate health information and shift social norms. Finally, at the structural level, The TALK addresses systemic barriers to HIV prevention by offering culturally tailored, accessible eHealth tools.

Through this multilevel approach, The TALK aims to strengthen resilience and support within Black male adolescents and young adults’ relationships and connection to the community to improve sexual health behavior and outcomes. By engaging parents, barbershops, and salons, the intervention empowers participants with shared language, nuanced communication skills, and tools to reduce discomfort associated with discussing sexual health.

### Aims and Objectives

Our objective is to develop the first nurse-led multipronged intervention, The TALK, which is a parent-centered, adolescent-involved health promotion intervention for Black male adolescents and young adults by using the following specific aims.

Aim 1: develop The TALK for use with fathers, mothers, and adolescent males in partnership with beauty industry partners and an established community advisory council.

Aim 2a: determine the usability, acceptability, and preliminary intervention outcomes of The TALK (frequency and quality of parent-adolescent sexual health communication, improvements in knowledge of HIV testing, improvements in parent-adolescent conversations around racial discrimination, improved perceived racial identity among Black adolescents, and decrease risky sexual behaviors).

Aim 2b: explore the use of signage in the beauty industry to promote and market the use of The TALK app (number of downloads, amount of usage and time in the app, etc).

This approach is innovative in that it uses a community engagement approach to address the intersectionality of racial discrimination and HIV risk transmission factors and has potential for wide-scale dissemination by using various real-world approaches.

## Methods

The TALK pilot is a nonrandomized study using convenience sampling.

### Ethical Considerations

This study was reviewed and approved by the Duke University School of Nursing institutional review board in September 2022 (Pro00105116). The informed consent documents will include detailed information on all study procedures as well as the consent process. Participating parents and sons will provide electronic consent prior to the start of the baseline survey and gaining access to the 6-week eHealth intervention. Each week, participants will receive electronic access to a new module with accompanying survey, assessment, and/or activity with postintervention surveys after completing the final module, which are all opt-in opportunities and conducted on a digital device with whatever privacy settings are chosen by those participants. All surveys will be collected and housed in the secure Research Electronic Data Capture (REDCap) platform to safeguard participant information [29,30]. Data are deidentified and then analyzed. Each parent participant will be compensated with a US $250 gift card.

### Participants

A representative group of fathers and mothers (n=60) along with their sons (n=60) will be recruited and engaged in The TALK intervention. The inclusion criteria for the study includes being a father (defined as biological father, stepfather, father figure, or father of an adopted son) or mother (defined as biological mother, stepmother, mother figure, or mother of an adopted son) of an adolescent son who identifies as Black/African American descent between the ages of 10 and 17 years. Parent and son dyads are included if both consent and agree to participate in The TALK over the 6-week period, including completing surveys, receiving research team communications, viewing resources and tools on The TALK on a weekly basis, and engaging in conversations and activities as instructed between parents and adolescents.

### Recruitment

We will use an inclusive approach for recruitment, meaning father and son, mother and son or father, or mother and son will be recruited for participation over the 6 weeks (n=60 fathers and mothers, n=60 sons). Barbershops and salons serving primarily Black customers will be identified throughout North Carolina through a telephone directory and registries of the State Board of Cosmetology and Board of Barber Examiners. Program managers will contact barbershops and salons via email, phone, face-to-face, or through social media to inform about the web-based application and provide signage to post in their shops.

### Intervention

The TALK intervention, a technology-based eHealth intervention delivered over 6 weeks, has 3 major components: (1) educational microlearning videos on various sexual health topics (eg, healthy relationships, sexually transmitted infections and HIV prevention, and pre-exposure prophylaxis), (2) 6 community-led podcasts that discuss sex, racial identity, and behaviors, and (3) an interactive parent-child activity using a deck of conversation cards to prompt dialogue (See Multimedia Appendix 1). Six electronic modules will offer content specific for parents, sons, as well as content for parents and sons to engage together. To allow individual and collective completion, modules will be released 2 at a time every 2 weeks. Communication is an essential component of healthy sexual development among Black male adolescents and young adults [31,32] and is a cornerstone of The TALK. To facilitate open communication between parents and Black male adolescents and young adults, each parental unit will receive a set of 30 conversation cards that spark conversation about relationships, consent, and perceived racial identity.

These health education tools are effective in promoting health, providing engaging health information and flexible delivery options [33-38]. Microlearning is an alternative method of educating that breaks down larger content to digestible short videos, infographics, or audio clips to increase engagement and has been shown to increase participant knowledge, self-efficacy, and self-care [39]. Various educational mediums offer several potential advantages such as providing consistency in the presentation and information delivered, reaching a broad audience, and video interventions can be a less resource-intensive means of delivering educational content [34,40,41]. Text messaging will also be used during the 6 weeks to alert users about certain contents and to access the app. Evidence suggests that inclusion of text messaging in health promotion interventions may lead to improved adolescent sexual health [42,43]. These technologies and a variety of engagement tools are combined to promote intervention adherence throughout the intervention.

### Strategy

There are 3 phases for this project: development, usability for community of interest, and testing for real world usability. In phase 1/aim 1, the development of The TALK, a parent-centered, community-engaged approach is utilized by examining idea validation of the intervention through the engagement of 13 representatives of fathers and mothers by using the dscout platform. Dscout is a digital platform for virtual ethnographic research used to explore any early-stage user experience issues and deliver glitches and validate the user view and need for the solution prior to launch and preliminary testing. This approach to product development reduces the risk of launching new products by testing and iterating the concepts in a rapid cycle way with potential users before investing larger amounts of time and money into a full product launch. A representative group of fathers and mothers (n=13) will be recruited and engaged in The TALK intervention through the dscout platform under the leadership and management of consultants hired on the project. This process in conjunction with meetings with members of the community advisory council will inform intervention development, including design layout and content.

In phase 2, we will recruit 60 dyads, consisting of a parent/child, for pretest-posttest survey data collection with a total of 120 participants to examine the usability, acceptability, and preliminary intervention outcomes of The TALK. This phase focuses on the frequency and quality of parent-adolescent sexual health communication, improvements in knowledge of HIV, improvements in parent-adolescent conversations around racial discrimination, and its impacts on sexual health and improved perceived racial identity among Black adolescents. Participants will engage in The TALK intervention over a 6-week period. A mixed methods design will assess the pretest-posttest changes along with single-time point measures that will assess intervention and module-specific outcomes.

In phase 3, we will examine the usability of the web-based modules of The TALK intervention in real-world experiences, while signage will be used to market The TALK in 10 barbershops throughout North Carolina after a 6-week concept pilot in aim 2. We will conveniently identify 10 barbershops and salons primarily serving Black customers throughout North Carolina. To do this, our program manager and beauty industry partners will contact shops via email, phone, face-to-face, using telephone directory or registries of the State Board of Cosmetology and Board of Barber Examiners, or through social media to inform them about The TALK and provide signage (with QR code to scan and access the website) to post in their shops if they agree. Limiting to 10 shops allows for control in comparing the characteristics of the shops (size, location, zip code, clientele, etc) and for considering barbershop characteristics as potential factors related to the real-world use of The TALK. Our previous studies and others have found that salon-based and barbershop-based interventions must consider shop characteristics for implementation, as there is no one size that fits all [44]. We will measure the following website metrics: page views, average time on page, average session duration, pages per session, bounce rate, and traffic sources.

### Data Collection

The measures table (Multimedia Appendix 2) summarizes each of the measures for phase 2 for analyzing the usability of the intervention. The TALK’s usability will be examined using the System Usability Scale (α=.90), which is a 10-item survey rating participant responses as strongly disagree to strongly agree [45]. Sample questions include “I think that I would like to use this frequently. I thought The TALK was easy to use.” Acceptability will be measured using the Acceptability of Intervention Measure (α=.85) [46,47]. This 4-item survey rates responses from completely disagree to completely agree. Sample questions include (1) The TALK meets my approval and (2) The TALK is appealing to me. A pre-post design will be used to examine the preliminary intervention outcomes of The TALK. Measures include improve frequency (Parent-Adolescent Communication Scale, α=.88) [48,49] and quality (Parent/Adolescent Communication-Jaccard, α=.93) [50-52] of sexual health communication, conversations about racism (Likert scale survey, principal investigator and consultant developed with validation assessed within this study), everyday experience of racial mistreatment (ecological momentary assessment, α=.80) [53], racial socialization (Racial Bias Preparation Scale’s subscales Reactive Messages, α=.86, and Proactive Messages, α=.83) [54], and perceived racial identity (Multidimensional Inventory of Black Identity Teen Scale Survey, 7 subscales, α=.75-.88) [55-57]. We will also obtain HIV and testing knowledge from both adolescents and parents. Baseline data will be collected in week 1 and poststudy data will be collected in week 6. We will also examine the perspectives of the participants on the use of video messaging (short clips) for the web-based application. Example questions will include: did the video clips provide you with tools to help you communicate more effectively with your son? What did you like most about the video clips?

Single-time point measures will be collected at the end of modules 2-5 to provide module-specific data with module 2 including a knowledge-based questionnaire (5-items) created by the researchers (validation within this study), module 3 including an engagement survey with usage and effectiveness questions (8 items) created by the researchers, module 4 including the Multidimensional Inventory of Black Identity Teen Scale [57], and module 5 including the Racial Bias Preparation Scale [54]. Open-ended qualitative questions were also asked after modules 4 and 5 to receive feedback from the participants about intervention content experience and use. Demographic data will be obtained from fathers, mothers, and adolescents and will include age, marital status, education, socioeconomic level, and does the child live in the home with one or both parents. We will use our Health Insurance Portability and Accountability Act–compliant research electronic data capture in REDCap to send out text messages with reminders to participants to complete surveys and engage in modules.

### Statistical Analysis

Demographic information will be summarized as the mean and standard deviation for continuous variables and frequency and percentage for categorical variables. Descriptive statistics for the intervention outcomes measures at each assessment point (week 1 and 6) and changes in these outcomes will be provided. Summary statistics will include n (%) for categorical measures and median (25th and 75th percentiles) along minimum and maximum values for continuous measures due to small sample sizes and expected skewness. For the continuous intervention outcomes, nonparametric Wilcoxon signed-rank tests for repeated measures will be used to test for change from week 1 to week 6 within each of the 3 participant groups (88 adolescents, 88 fathers and mothers). Nondirectional tests will be performed with the significance level set at .05 to increase statistical power based on the assumption that small-to-moderate change effect sizes within each group will be observed.

## Results

This project was funded by the Gordon and Betty Moore Foundation and approved by the Duke University School of Nursing institutional review board in September 2022 (approval Pro00105116) for development with the community. Starting October 2022, intervention components were developed in partnership with community partners. User feedback on initial website design, videos, and comment cards were provided by parents of Black male adolescents and young adults (n=13) by using the dscout platform. Then, the intervention content was finalized using a community-engaged iterative process involving community members, stakeholders, and businesses. This collaborative approach was overseen by the research team and our community advisory board. Data collection for phase 2 began in July 2023 and ended in January 2024. Data analysis is scheduled for completion by July 2025. A manuscript on the intervention design process and findings is under review. The publication of the primary and secondary results is expected by January 2026.

## Discussion

### Anticipated Findings

This paper presents a protocol to deliver a culturally responsive and influenced sexual health promotion intervention specifically designed for Black parents and their adolescent sons. We anticipate that The TALK intervention will be feasible and acceptable, improve parent-adolescent communication, and improve knowledge of HIV and sexually transmitted infections among Black adolescent males. Additionally, this study will provide evidence that builds on growing literature contributing to digital interventions to improve sexual health and HIV education and communication among Black adolescents. Implementation of The TALK protocol will allow the authors to link their findings from a sample of Black parents and sons with the evidence of prior studies among Black mothers and daughters to better understand family relationship dynamics in regard to racial and sexual conversations. The TALK’s structure, content, and utilization of technology was developed with feedback from beauty industry partners and community members with the goal of improving feasibility and acceptability of the intervention. These community-engaged steps are expected to improve the overall impact and outcome of the intervention. As a result, the goal is to influence future research in family communication about sexual health and HIV.

### Strengths and Limitations

The design of this study has several strengths, including its focus on Black adolescent males and their parents/guardians, which has the potential to improve the shared knowledge and communication within the parent-adolescent relationship and influences Black adolescent males’ behavior and health outcomes. The research team is experienced in nurse-led, community-engaged research and health promotion interventions using various technology-based methods, which improves intervention and research design and implementation. The sample size and analysis by different demographic characteristics can provide insights into the influence of similarities and differences on the changes experienced after the intervention by participants. The use of the dscout platform to develop and beta test the intervention can increase the cultural relevancy and appeal of an intervention throughout the stages of development. Previous community-based collaborations and research by the study team with Black communities have informed that the intervention’s content and method design can impact participant recruitment and intervention relevance for the target population, which is promising for future research and broader impact on Black male adolescent health behavior and outcomes as well as the design of culturally relevant interventions. The anticipated limitations of this intervention include the self-report of improvements in communication and health behaviors (eg, sexually transmitted diseases, HIV testing). The intervention includes multiple electronically delivered modules that are prone to participant attrition, which will demand counter recruitment and retention efforts to maintain sufficient parent-adolescent pairs of complete data.

### Future Directions

The findings of this study will be disseminated to the study’s participants in a brief newsletter authored by the research team, social media posts (eg, video blogs, short posts), as well as in a lay community journal. Additionally, presentations will be given to the local Black community at local and regional community events. The findings will be disseminated to the scientific community by using multiple strategies such as presentations at national and international HIV conferences, publications in nursing and interprofessional peer-reviewed clinical and research-based journals, and media press releases. The intention to disseminate to scientists, clinicians, and the community will contribute to the science of behavior change.

To prepare for scalability, The TALK will expand its real-world use and application by becoming an available resource accessible in barbershops and salons across the southern region. There is a future need to examine the long-term impact of TALK on parents’ communication engagement and sexual health outcomes of adolescents over time. Opportunities also exist for future engagement of The TALK as a resource for health care providers in clinical encounters. Linking culturally sensitive and relevant messaging in health care settings for parents and adolescents contributes to more effective communication. This can help bridge cultural gaps and make it easier for providers to discuss sensitive topics such as sexual health or the impact of lived experiences on health and well-being.

### Conclusion

Disparities in HIV infections for Black adolescents and young adults persist despite an overall decrease in HIV incidence. Addressing Black male adolescents and young adults’ perceived racial identity and primary social influences is integral to creating Black male adolescents and young adults’ social norms around sexual behavior. The TALK offers a culturally responsive, community-informed approach to engaging parents and Black male adolescents and young adults in sexual health promotion strategies that normalize communication, increase individual sexually transmitted infection/HIV prevention behaviors, and could be used to influence pre-exposure prophylaxis use.

## Appendix

Multimedia Appendix 1 The TALK intervention components.

Multimedia Appendix 2 Study measures.

## Abbreviations

REDCap: Research Electronic Data Capture
