# Supplementary material for: A Multipronged, Community-Partnered Intervention (The TALK) to Improve Parent-Adolescent Communication About Sexual Health and Racial Discrimination Among Black Male Adolescents and Young Adults and Their Caregivers: Protocol for a Feasibility and Acceptability Study
Source: JMIR Res Protoc. 2025 Jul 8;14:e67403. doi: 10.2196/67403 (PMC12284450; doi:10.2196/67403)
Supplement: Multimedia Appendix 1 [file resprot_v14i1e67403_app1.doc]

**Appendix A: *The TALK* Intervention Components**

| **Component** | **Description** | **Goals** | **Target Audience** | **Implementation Strategy** |
| --- | --- | --- | --- | --- |
| Conversation Cards | Cards with prompts to facilitate discussions on sexual health topics | Encourage open dialogue between adolescents and caregivers to Increase awareness, engagement, and communication. | Adolescent and Caregiver | Distributed in barbershops and salons |
| Community-led Podcast | A podcast series featuring community members discussing sexual health | Provide relatable information, foster community engagement, and share experiences through stories. | Caregiver | Partner with local barbers and salons  Promote through social media and local events |
| Web-based Sexual Health Modules | Online interactive modules cover various aspects of sexual health, including HIV prevention, PrEP medication, and contraception. | Provide accurate, high quality sexual health information along with strategies for interpersonal relationships and communication. | Adolescent and Caregiver | Accessible in Barbershops via a QR code or shared via social media sites |
