# Supplementary material for: A Multipronged, Community-Partnered Intervention (The TALK) to Improve Parent-Adolescent Communication About Sexual Health and Racial Discrimination Among Black Male Adolescents and Young Adults and Their Caregivers: Protocol for a Feasibility and Acceptability Study
Source: JMIR Res Protoc. 2025 Jul 8;14:e67403. doi: 10.2196/67403 (PMC12284450; doi:10.2196/67403)
Supplement: Multimedia Appendix 2 [file resprot_v14i1e67403_app2.doc]

**Appendix B: Study Measures**

| **Measurement** | **Measurement Description** | **Cronbach’s alpha** | **Expected Outcome** | **Source** | **Evaluation Schedule** |
| --- | --- | --- | --- | --- | --- |
| Systems Usability Scale (SUS) | A 10-item measure of digital intervention usability. Items are scored on a 5-item Liker scale (Strongly Disagree-Strongly Agree) [46]. | α = 0.90 | Usability | Father  Mother | Post-Intervention |
| Acceptability Intervention Measure (AIM) | A 4-item measure of perceived intervention acceptability for *The TALK* web-based application. Items are measured on a 5- point Likert-scale (Completely Disagree-Completely Agree). Score is calculated as mean [47,48]. | α = 0.85 | Acceptability | Father  Mother Adolescent | Post-Intervention |
| The Parent/Adolescent Communication- Jaccard (PAC-J) | A 16-item scale for (adolescents) and 21-item for (parents) assessing the quality of communication between an adolescent and his parents concerning issues of sex. The scale was validated on a sample of 751 African American adolescents between the ages of 14 to 17 [51-53]. | α = 0.93 | Improve the quality of parent-adolescent sexual health communication | Father  Mother  Adolescent | Pre-Post Intervention |
| Parent-Adolescent Communication Scale (PACS) | *A* 5-item scale designed to assess adolescents’ self-reported frequency of communicating about sexually related topics with their parents. Adolescents and parents will report on the frequency of their communication about sexual health [49,50]. | α = 0.88 | Improve frequency of sexual health communication | Father  Mother  Adolescent | Pre-Post Intervention |
| Multidimensional Inventory of Black Identity –Teen Scale | A 21-item scale to understand the heterogeneity in African Americans’ attitudes regarding the importance and meaning that they attach to race. There are 7 subscales with 3 categories of questions, including racial centrality, regard, and ideology [58]. | Centrality (8 items; α = .78); Private Regard (6 items; α = .87); Public Regard (6 items; α = .79); Nationalist (9 items; α = .87); Assimilationist (9 items; α = .88); Humanist (9 items; α = .75); Minority (9 items; α = .80) | Improved perceived racial identity | Adolescent | Pre-Post Intervention |
| Racial Bias Preparation Scale | A 30-item self-report inventory designed to assess youth perceptions of racial socialization; assess the frequency with which youth perceive the reception of messages from their primary caregivers regarding racial experiences. Participants respond to questions on a 3-point Likert scale from *never* (1) to *a lot* (3) [55]. | Reactive Messages α = 0.86 and Proactive Messages α = 0.83 | Explore changes in Perceptions of Racial socialization | Adolescent | Pre-Post Intervention |
| Experience with Everyday Discrimination | A 5-item survey to explore the experiences of racism in everyday life. Responses include almost every day, once a week, few times a month, few times a year, less than once a year, never [54]. | α = 0.80 | Sample Characteristic | Father  Mother  Adolescent | Pre-Post Intervention |
| Knowledge Scale | A 5-item original measure to assess general knowledge of sexual health and HIV information shared within intervention Module 2. | Validation within this study. | Knowledge | Father  Mother  Adolescent | Post-Module 2 |
| Comment Card Survey | An 8-item original measure to assess Comment Card activity engagement, including items addressing Comment Card usage and effectiveness. | Validation within this study. | Engagement | Father  Mother  Adolescent | Post-Module 3 |
| Open-ended questions | Questions focused on feedback related to intervention content and use. |  | Feedback | Father  Mother  Adolescent | Post-Modules 4 and 5 |
